# Insight from *OPN1LW* Gene Haplotypes into the Cause and Prevention of Myopia

**DOI:** 10.3390/genes13060942

**Published:** 2022-05-25

**Authors:** Maureen Neitz, Melissa Wagner-Schuman, Jessica S. Rowlan, James A. Kuchenbecker, Jay Neitz

**Affiliations:** 1 Department of Ophthalmology, University of Washington, Seattle, WA 98109, USA; jsrowlan@uw.edu (J.S.R.); jkuchen@uw.edu (J.A.K.); jneitz@uw.edu (J.N.); 2 Department of Psychiatry, University of Illinois at Chicago, Chicago, IL 60612, USA; wagnermu@ucmail.uc.edu

**Keywords:** myopia genetics, cone opsin genes, Xq28, exon skipping, splicing mutation

## Abstract

Nearsightedness (myopia) is a global health problem of staggering proportions that has driven the hunt for environmental and genetic risk factors in hopes of gaining insight into the underlying mechanism and providing new avenues of intervention. Myopia is the dominant risk factor for leading causes of blindness, including myopic maculopathy and retinal detachment. The fundamental defect in myopia—an excessively elongated eyeball—causes blurry distance vision that is correctable with lenses or surgery, but the risk of blindness remains. Haplotypes of the long-wavelength and middle-wavelength cone opsin genes (*OPN1LW* and *OPN1MW*, respectively) that exhibit profound exon-3 skipping during pre-messenger RNA splicing are associated with high myopia. Cone photoreceptors expressing these haplotypes are nearly devoid of photopigment. Conversely, cones in the same retina that express non-skipping haplotypes are relatively full of photopigment. We hypothesized that abnormal contrast signals arising from adjacent cones differing in photopigment content stimulate axial elongation, and spectacles that reduce contrast may significantly slow myopia progression. We tested for an association between spherical equivalent refraction and *OPN1LW* haplotype in males of European ancestry as determined by long-distance PCR and Sanger sequencing and identified *OPN1LW* exon 3 haplotypes that increase the risk of common myopia. We also evaluated the effects of contrast-reducing spectacles lenses on myopia progression in children. The work presented here provides new insight into the cause and prevention of myopia progression.

## 1. Introduction

Myopia is a leading cause of blindness [1,2,3], and its prevalence is rising [2,4,5]. Genetic and environmental factors contribute to excessive elongation of the eye, which causes myopia [6]. Despite intensive searches for myopia genes in genome-wide association studies (GWAS), identified markers explain only ~18.4% of the heritability and 12.1% of the variance in spherical equivalent refraction (SER) [7,8,9,10]. Moreover, GWAS results have not translated into new prevention strategies. GWAS identify markers that can predict the presence of a disease gene but rely on the candidate gene approach to identify the nearest probable causative gene without significant knowledge of the mechanism by which the candidate genes cause the disease.

Polymorphisms in the human long-wavelength- (L) and middle-wavelength-sensitive (M) cone opsin genes (*OPN1LW* and *OPN1MW*, respectively) are associated with myopia [11,12,13,14,15,16], and they exhibit high haplotype diversity [17,18,19], making them good candidate genes for involvement in common myopia. Furthermore, the polymorphisms underlying high myopia have led to the discovery of the genetic mechanism by which these genes cause myopia. High-myopia-associated haplotypes induce 80–100% of exon-3 skipping in vitro [11,12,13,14,15,20,21], and in vivo, they give rise to cone photoreceptors that are nearly devoid of photopigment. These cone photoreceptors expressing mutant opsin are randomly distributed in the retinal mosaic, with cones expressing “normal” amounts of photopigment [16,22,23]. Having adjacent cones with vastly different amounts of photopigment due to strong exon-3 skipping is associated with high myopia [16]; thus, we looked for an association between weaker exon-3-skipping haplotypes and common myopia.

Genetic analysis of the *OPN1LW* and *OPN1MW* genes is complicated by many factors. The genes are arrayed in tandem on the X-chromosome with a high degree of copy-number variation [24,25]. Most males have at least three opsin genes, and most females have at least six [26,27]. Moreover, the *OPN1LW* and *OPN1MW* gene sequences are nearly identical, and only the first two genes in the array are expressed [28]. Thus, the *OPN1LW* and *OPN1MW* genes are intractable to investigation via the methods typically employed in GWAS or other high-throughput methods. Steps must be taken to ensure that the gene being interrogated for association with disease is one of the two expressed opsin genes. Thus, we investigated the role of the Xq28 cone opsin genes in myopia by sequencing the *OPN1LW* gene and estimating spherical equivalent refraction (SER) from the axial length and corneal curvature measurements for males with normal color vision. Such males typically have one *OPN1LW,* followed by one or more *OPN1MW* genes; thus, by restricting the analysis to the *OPN1LW* gene first in the array, we can be certain the gene being studied is expressed [28]. We report that *OPN1LW* SNPs are significantly associated with common myopia.

Due to their receptive field circuitry, a subset of cones with significantly lower amounts of photopigment due to exon-3 skipping might produce spurious contrast. Abnormal contrast signaling may stimulate axial elongation. We describe a pilot study showing that contrast-reducing eyeglasses effectively slowed the axial elongation rate in myopic children. A more optimized version of the spectacles reduced myopia progression by 74% after one year in a controlled, randomized, multisite clinical trial (ClinicalTrials.gov Identifier: NCT03623074) [29]. Combining opsin genetics and myopia-progression-reducing eyeglasses provides a rational approach to control eye growth safely and effectively with the potential to reduce the population risk for blindness due to complications of myopia [6].

## 2. Materials and Methods

### 2.1. Cell Lines

Authenticated HEK 293T (female) cells (RRID: CVCL_0063) were obtained from ATCC. Cells were cultured in Dulbecco’s Modified Eagle’s Medium (DMEM) supplemented with 10% heat-inactivated fetal bovine serum and 2 mM L-glutamine and maintained at 37 °C with 95% carbon dioxide.

### 2.2. Human Subjects

Three hundred and seventy-three adult subjects provided their recent refractive error measurements from their optometrist or ophthalmologist. Study procedures for these subjects included color vision testing using the Richmond HRR pseudoisochromatic plates (4th edition) and measurements of axial lengths and corneal curvatures using the Zeiss IOL Master. We derived an equation that estimates each participant’s spherical equivalent refraction (SER) from the IOL Master data (Supplementary Figure S1).

We recruited 1000 adult males and identified 413 that qualified for the analysis to evaluate the relationship between opsin gene haplotypes and SER based on inclusion criteria. Subjects were asked to report their ancestry and were only included if they reported being of European ancestry to avoid population stratification in the genetic analysis. Participants were asked to perform a color vision test and were only included in the analysis if the test results indicated normal trichromatic color vision. Participants were excluded if they had prior eye surgery or eye injury as these can affect refractive error. Additional procedures for these subjects included measurements of axial lengths and corneal curvatures using the Zeiss IOL Master, collection of blood or saliva samples for DNA extraction, and flicker-photometric electroretinography (ERG) [18]. We sequenced exons 2, 3, and 4 of the *OPN1LW* and *OPN1MW* genes separately using gene-specific amplification followed by Sanger sequencing (described in more detail below) [30]. We estimated the relative number of *OPN1LW* and *OPN1MW* genes on the X-chromosome for each subject using the Agena MassArray genotyping system (described in more detail below) [27]. The ratio of L to M cones for each subject was estimated from the ERG spectral sensitivity data and genetic data, using previously described methods [18]. Subjects were included in the analysis only if they had a single *OPN1LW* gene sequence to ensure analysis of an expressed opsin gene since only the expressed opsin genes can contribute to refractive error development. To calculate the mean SER for each haplotype, we only included individuals with an *OPN1LW* haplotype shared by at least four participants. Ultimately, 413 subjects fit the inclusion criteria for the analysis (Table 1, Supplementary Figure S2).

We tested contrast-reducing eyeglasses for their effect on myopia progression in 13 children. We obtained approval for the eyeglasses study from the IRB at the Medical College of Wisconsin and Children’s Wisconsin Hospital Milwaukee. Procedures performed on participants included color vision testing with the HRR Richmond 4th edition pseudoisochromatic test, axial lengths, and corneal curvature measurements made using the Zeiss IOL Master. Inclusion criteria included that children had to be in the early stages of rapidly progressing myopia and had progressed at least −1.0 diopters in the year prior to enrolling in the study. Participants (*n* = 13) were referred by eye care practitioners who knew the eligibility requirements. Thus, all subjects we assessed were eligible. The 13 participants enrolled for 3 months and were allowed to re-enroll for a second 3 months. Seven participants elected to re-enroll for the second 3 months of the study. The characteristics of each participant are listed in Supplementary Table S1. All subjects completed the first 3 months, and seven completed the second 3 months. No participant withdrew from the study. All data from all subjects were included in the analysis.

Axial lengths and corneal curvatures were measured for each subject using the Zeiss IOL Master. The spherical equivalent refractions (SERs) were calculated using a formula derived from a linear regression of a dataset of observed SERs, axial lengths (AL), and corneal curvatures (CC) from a group of 373 subjects (Figure S1). The formula is: SER = −(AL × 2.03 + 0.94 × CC) + 88.58, where the value for AL was the average of 20 measurements per eye, and CC was the average of the two different methods of measuring read-outs from the IOL master.

### 2.3. Opsin Gene Sequencing

We specifically sequenced exons 2, 3, and 4 of the *OPN1LW* and *OPN1MW* genes by first using the polymerase chain reaction (PCR) to specifically amplify a segment of the *OPN1LW* and *OPN1MW* genes that included exons 2 through 5. Exon 5 encodes amino acid differences that are responsible for the majority of the spectral difference between L and M cones, and thus the sequence of exon 5 functionally distinguishes between *OPN1LW* and *OPN1MW* genes [30,31]. Reverse primers selective for *OPN1LW* exon 5 or *OPN1MW* exon 5 were paired with a non-specific intron 2 primer to separately amplify exons 2 thru 5 of the *OPN1LW* and *OPN1MW* genes. These long-distance PCR products were used in nested PCR to amplify the individual exons and ~50 bp of the flanking introns. These exons and the intron/exon junctions were directly sequenced by fluorescent Sanger sequencing using BigDye Terminator cycle sequencing. The PCR and sequencing primers and thermal cycling conditions have been described in detail previously [30].

### 2.4. Minigenes

We created minigenes using the procedure described by Ueyama et al. [20]. Minigenes contained a full-length human *OPN1LW* cDNA with the full-length introns 2 and 3 inserted on either side of exon 3. Site-directed mutagenesis was performed using the QuickChange kit (Agilent) or the In-Fusion system (Clonetech) to create 128 different exon 3 haplotypes which were cloned into the minigene using the HindIII and AflII sites flanking exon 3. The 128 minigenes generated were sequence-verified, and the only differences among them were the sequences of exon 3. Exon 3 haplotypes varied at eight nucleotide positions, described here using SNP ID numbers: rs949930 was A or G, rs713 was A or C, rs731614 was C or G, rs5986963 was A or G, rs5986964 was T or G, rs149897670 was C or T, rs145009674 was A or G, and rs155715655 was G or T. Minigenes represented all combinations of these SNPs except rs5986964. Typically, in *OPN1LW* genes, rs5986963 and rs5986964 are A and T, or G and G, so these are the only combinations represented in the 128 minigenes. Exons 2 and 4 of the human *OPN1LW* gene are also polymorphic. The minigenes all had the following residues at the variable positions: c. 194C (rs782815809) c.300A (rs1065420), c.331A (rs1065421), c.347C (rs1065422), c.689T (rs148583295), c.697G (rs781936473), c.698C (rs1292153674), c.699T (rs1226772901), and c.706A (rs1065426).

### 2.5. Splicing Assays 

Using the procedure described by Ueyama et al. [20], 1.1 µg of each minigene plasmid was transfected into duplicate cultures of HEK293T cells using Fugene HD. Forty-eight hours after transfection, RNA was isolated, reverse transcribed using the Superscript First-Strand Synthesis kit (Thermofisher), and primed with oligo dT. The resulting cDNA was used in a genotyping assay employing the Agena MassArray genotyping platform to estimate the percentage of cDNA lacking exon 3 for each of the 128 exon 3 haplotypes.

### 2.6. The MassArray Assay 

The MassArray genotyping platform was used for the quantitative assessment of exon-3 skipping. The assay was first validated by creating a standard curve using known ratios of DNA templates with exon 3 included and exon 3 excluded. Genotyping was performed using the Agena iPLEX Pro kit according to the manufacturer’s instructions. Briefly, cDNA was subjected to PCR amplification and primer extension using the iPLEX Pro PCR reagents. Assays, including primers, were designed using the manufacturer’s software. A cDNA segment extending from exon 2 to exon 4 was PCR-amplified with a forward primer to sequences in exon 2, starting 73 bp upstream of the 3′ end of exon 2 (primer sequence 5′ ACGTTGGATGAACCAGGTCTCTGGCTACTT) and a reverse primer to sequences in exon 4 starting 82 bp downstream of the 5′ end of exon 4 (reverse primer sequence 5′ ACGTTGGATGAATCTCACATTGCCAAAGGG). The underlined bases in each primer were added to remove the primers from the mass window observed in the analysis of the extension products by the MassArray MALDI-TOF mass spectrometer. The PCR reactions were treated with iPLEX Pro Shrimp Alkaline Phosphatase to dephosphorylate the unincorporated dNTPs. The PCR products were then subjected to a single-nucleotide primer extension reaction using the iPLEX Pro Extend reagents. The extension primer sequence was 5′ AGGCCGTGGGGCCAGTACC, which corresponds to the 5′ end of exon 4 extending toward exon 3 with the last nucleotide of the primer corresponding to the 3′-most nucleotide of both exons 2 and 3. The extension reaction incorporates a T residue if exon 4 is spliced to exon 3, or an A residue if exon 4 is spliced to exon 2 and exon 3 is skipped. Extension reactions were carried out with mass-modified terminator dNTPs so that the T vs. A difference in the extension products was sufficient to resolve them by MALDI-TOF mass spectrometry. Desalting of the extension reactions was accomplished by adding iPLEX Clean Resin, followed by spotting reaction products onto a SpectraChip (Agena), and analyzed with the MassArray MALDI-TOF mass spectrometer. Note, if intron 2 had been retained, the extension product would have been the same as that expected if exon 2 had been spliced to exon 3 and would have lead to an underestimate of the amount of exon 3 skipped. However, PCR products were observed by agarose gel electrophoresis and subjected to Sanger sequencing. The major products were correctly spliced and exon-3-skipped; thus, intron retention is unlikely to affect the results significantly. The areas under the curve (AUC) for each of the extension products were used to calculate the percentage of mRNA with exon 3 skipped (% exon 3 skipped = 100 × (area under A extension product)/(area under A extension product + area under T extension product). The percentage of exon-3 skipping for each haplotype was taken as the average of the number of biological replicates performed, typically two replicates (see Supplementary Table S2). The within-group standard deviation is typically 1.5% or less with this assay; thus, using duplicate biological replicates provided 80% power to detect a 5% difference in exon-3 skipping with a *p*-value of 0.05.

### 2.7. Statistics

GraphPad InStat 3.06 was used to perform statistical analyses, except for the split-halves analysis, which was done using a custom MATLAB script.

The initial analysis to test for an association between the calculated SER and the haplotype of *OPN1LW* exon 3 was performed using the Kruskal–Wallis non-parametric ANOVA because many of the haplotype groups did not follow a Gaussian distribution, nor did they have identical standard deviations. The split-halves analysis was performed using data from the same subjects randomized into two groups. Haplotypes in each group were ranked according to their average refractive errors, with haplotype rank 1 having the most myopic refractive error and rank 11 having the least myopic refractive error. In the absence of an association between haplotype and refractive error, the correlation between rank and refractive error would not be statistically significant. Because the results for any two halves are independent, the correlation between ranks for the two halves would also not be statistically significant. To obtain the most accurate ranking possible for the haplotypes, we repeated the random-draw ranking 1000 times, then averaged the rankings of the 1000 randomized halves. We validated the procedure by repeating the process with the same subjects and the same haplotype group sizes, but we randomly scrambled the assignments of the subjects to haplotype groups. This yielded a non-significant *p*-value of 0.44 and a low *r*^2^ of 0.07, demonstrating that the procedure does not produce an artifactually significant correlation.

The minigene splicing results were analyzed using the Mann–Whitney U non-parametric test because the assay results were not normally distributed. For each SNP evaluated, the results of the splicing assay were compared for 64 minigene pairs that differed by a single nucleotide.

## 3. Results

### 3.1. The Relationship between Xq28 Opsin Gene Haplotypes and Refractive Error

**Figure 1:**
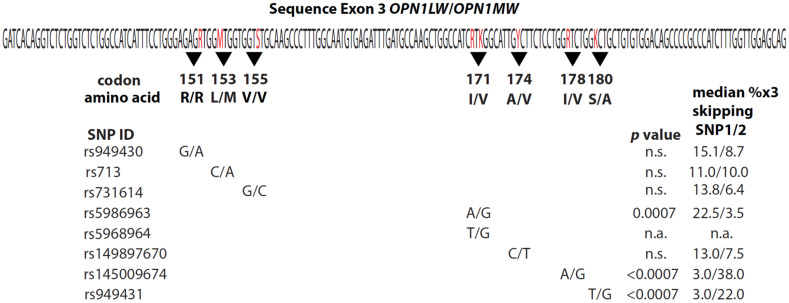
Four of seven exon 3 SNPs differ significantly in exon skipping The nucleotide sequence of *OPN1LW/OPN1MW* exon 3 is given. The eight polymorphic nucleotides analyzed in this study are shown in red letters using the IUB code where R is A or G, M is A or C, S is G or C, K is G or T, and Y is T or C. Black triangles underscore the codons containing the SNPs and the codon numbers are indicated. More information about each SNP can be found at in dbSNP (https://www.ncbi.nlm.nih.gov/snp/, accessed on 1 February 2022). Below the codon numbers are the two alternate amino acids encoded, below which are the two common nucleotides at each SNP. At each position, the amino acid and nucleotides are given as allele 1/allele 2. For example, codon 151 is either AGG or AGA, designated G/A. Both of these codons encode arginine, designated R/R using the single-letter amino acid code. Codon 153 is either CTG or ATG, designated C/A, and encodes leucine or methionine, respectively, designated L/M (see Table 1 for the single letter amino acid code). SNP ID numbers in the left-most column are from dbSNP151 for the *OPN1LW* gene. The median percentage of exon-3 skipping for each SNP except the third nucleotide of codon 171 (labeled n.a. for not applicable) is in the right-most column. A slash (/) separates values for alleles 1 and 2. After adjusting for multiple comparisons by applying the Bonferroni correction, three of the SNPs were significantly associated with exon-3 skipping (n.s. indicates adjusted *p*-value was not significant).

The relationship between SER and *OPN1LW* haplotypes was evaluated using 413 adult males with normal color vision [27,30,32] (see methods). Sequences and relative numbers of *OPN1LW* and *OPN1MW* genes were determined [27,30]. We calculated each subject’s SER from axial lengths, and corneal curvatures (raw data available upon request) using the equation derived in Supplementary Figure S1. There were 11 haplotypes among the 413 participants, and the median SER varied significantly more than expected by chance (*p* = 0.0082, Kruskal–Wallis test), indicating a role for *OPN1LW* polymorphism in common myopia; however, there were too many haplotypes to identify specific myopia-susceptible haplotypes via multiple comparisons. To determine this, we tested for an association between calculated SERs and the exon 3 SNP that most significantly impaired splicing in a minigene splicing assay. The percentage of exon-3-skipped mRNA was measured for 128 minigenes with different exon 3 haplotypes (Supplementary Table S2). Splicing was most impaired for exon 3 haplotypes with G at SNP rs145009674 in codon 178 (Figure 1), which skipped exon 3, on average, 12.7 times more than haplotypes with A (*p* < 0.0007 after correcting for multiple comparisons). SNPs rs155715655 in codon 180 and rs5986963 in codon 171 also significantly impaired exon-3 inclusion, but to lesser degrees (Figure 1).

Comparing the SERs calculated for subjects with A (*n* = 401) versus G (*n* = 12) at rs145009674 revealed a median SER of −0.9975D for individuals with A and −2.920 D for individuals with G; thus, the median effect size was ~2 D (*p* = 0.005, Mann Whitney U). The mean calculated SER for the two groups differed by 1.36 D (−1.1486 vs. −2.504), establishing this SNP in *OPN1LW* as significantly associated with common myopia. The population frequency of G at SNP rs145009674 is ~2.5%, and among Europeans, it is 2.07% [33]. In our sample, the calculated SER was worse than −0.75D for 82% of individuals with G at rs145009674. Reportedly, 30 out of 100 Europeans are myopic [34]. If 2 or 3 of 100 Europeans have G at rs145009674, 82% of them (1.7 to 2.5 people) with the G allele will be among the 30 myopes. Thus, having G at rs145009674 is associated with about 6–8% of myopes. The combination of a large effect size (about 2 D) and high frequency in the population makes this single SNP a significant determinant of common myopia.

We noted above that two additional SNPs are associated with significant exon-3 skipping. The median SERs for the 11 common haplotype groups varied more than expected by chance, and the mean SERs ranged over more than 4 D (Table 1). This suggests that the *OPN1LW* haplotypes are responsible for more refractive error than is evident just from our analysis at SNP rs145009674, but how much? About half of the refractive error SNPs discovered by GWAS are associated with myopia, accounting for ~6% of the myopic variance [8]. We estimated the variance in SER attributable to *OPN1LW* polymorphisms by ranking the myopia risk of the haplotypes using a split-halves analysis (Table 1, see methods). The mean refractive errors of the haplotypes were strongly correlated to the final haplotype ranks (Supplementary Figure S2) with a coefficient of determination (*r*^2^) of 0.98; the probability of this occurring by chance is 4.51 × 10^−9^. Haplotype rank can be used as a measure of the relative risk an individual has for becoming myopic. Thus, the degree of association, as indicated by calculating the Pearson’s correlation coefficient, between SER and haplotype rank for individual subjects is a gauge for the amount of variance in myopia attributable to the *OPN1LW* haplotypes; the coefficient of determination for this association is 0.046, indicating polymorphisms in *OPN1LW* account for about 4.6% of the variance in common myopia.

We could only analyze the 11 *OPN1LW* haplotypes found in at least four subjects, which reduced our sample size. However, lower-frequency *OPN1LW* haplotypes and *OPN1MW* haplotypes are likely to contribute to myopia through a similar mechanism. For example, in our sample, males with *OPN1MW* genes encoding only MVVVA (*n* = 64) versus everybody else (*n* = 349) differed in average SER by 0.39 D (raw data available upon request). Adding this single *OPN1MW* polymorphism to the opsin risk calculation raises the *r*^2^, and thus the amount of variance explained by polymorphisms at the X-chromosome opsin gene locus, to 4.9%. Determining how much of the variance in SER is attributable to the full spectrum of haplotypes at the *OPN1LW*/*OPN1MW* locus would require a larger sample. However, this study revealed that haplotypes of these genes are the major heritable risk factors for myopia.

We hypothesized that signals generated by having a lower amount of photopigment expressed in a submosaic of cones as a result of expressing an exon-3 skipping haplotype stimulates excessive eye growth [35]. If so, people with skewed ratios of L to M cones (L:M) should be relatively protected from myopia because cones with different abilities to catch photons are less likely to be adjacent to each other. Red–green color blind individuals have the most skewed cone ratios, having 100% L or 100% M cones, and are relatively protected from myopia [36]. We compared color-normal subjects with skewed cone ratios to those with more balanced proportions. The average cone ratio in people of European ancestry is about 2L:1M [37,38]. Thus, we determined the cone ratios in 357 of the 413 subjects (raw data available upon request) using previously described methods [37] and split the sample into a “skewed group” (≥67% and ≤33% L cones, *n* = 164) and a “more balanced group” (≤66% and ≥34% L cones, *n* = 193). The skewed group had, on average, 0.61 D less myopia (*p* = 0.006) than the balanced group. Having a more balanced ratio of L to M cones is, thus, a significant risk factor for myopia.

### 3.2. Myopia Prevention Using Spectacles That Control the Spatial Distribution of Light Reaching the Retina

**Figure 2:**
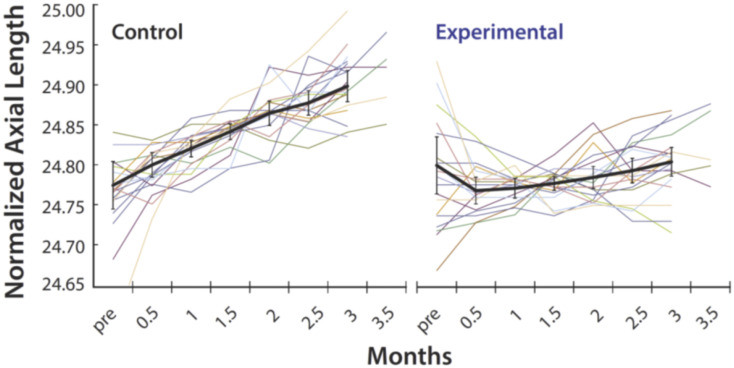
Normalized axial length measurements as a function of time. Thirteen dominant eyes wore the experimental lens (**right**), and 13 fellow eyes wore the control lens (**left**) for 3 months. Seven subjects re-enrolled for a second three-month period, during which the non-dominant eye wore the experimental lens and the dominant eye wore the control lens. Myopia progression significantly slowed in both groups (13 eyes and 7 eyes) when the two groups were analyzed separately. The combined data are shown. The data points for each subject are connected by a colored line. Each colored line represents measurements for one eye. Lines of the same color in the right and left plots represent data from the same subject for the eye wearing the control lens (**left**) and for the eye wearing the experimental lens (**right**). Black lines are averages for all eyes wearing the control lens (**left**) or wearing the experimental lens (**right**). Error bars are ± 2 SEM. The experimental lenses significantly reduced the rate of eye growth of myopic children (*p* = 0.001). Each data point is an average of twenty measurements.

The association between high myopia and exon skipping in the Xq28 opsin genes led us to propose the unorthodox hypothesis that increased contrast signaling is the common pathway in myopia development and progression. We tested the theory by measuring the effect of reducing retinal contrast on axial elongation in children with progressive myopia. To maximize statistical power and minimize sample size, each participant wore an experimental lens on one eye and a control lens on the other eye. Both lenses were standard-of-care single-vision lenses. The experimental lens had a red-light blocking tint and light scattering elements that spread incident light over a 0.5-degree angle, reducing contrast and the relative activity difference between adjacent cones that results from visual experience. The best-corrected acuities of participants ranged from 20/15 to 20/20 without light scattering and 20/20 to 20/25 with light scattering. The control lens had a neutral tint, so both eyes experienced the same luminance, removing it as a variable. Participants were in the early stages of rapidly progressing myopia (Table S1), documented by a change in refractive error of at least −1.0 D during the previous year. Thirteen subjects wore the experimental lens on their dominant eye and the control lens on the non-dominant eye. After 3 months, seven participants re-enrolled for another 3 months, during which they wore the experimental lens on the non-dominant eye and the control lens on the dominant eye. Supplementary Table S2 lists the baseline characteristics of participants. The primary outcome measure was the change in axial length from baseline for the eye wearing the experimental lens versus the control lens over 3 months. As predicted by the contrast theory of myopia, Figure 2 shows that there was a dramatic reduction in the average axial length growth rate in the eyes wearing the experimental lens (0.063 ± 0.33 µm/day; mean ± SE), versus control (1.43 ± 0.24 µm/day), and the difference was highly statistically significant (*p* = 0.0019). The growth rate of the control eyes in this study is larger than that reported for studies involving more children and lasting 1 to 3 years [39]. This is due to our inclusion criteria requiring very fast progressing myopes and the short duration of our study.

## 4. Discussion

### 4.1. Implications of Xq28 Opsin Gene Haplotypes for Females

We studied males but expect that a significant amount of myopia is associated with *OPN1LW* haplotypes in females; however, demonstrating this requires a larger sample size because females have two X-chromosomes while males have one. The observation that having a submosaic of mutant cones intermixed with normal ones in males is associated with myopia predicts that *OPN1LW* haplotypes manifest as X-linked dominant for myopia, consistent with reports that female carriers of the LVAVA cone opsin variant are myopic [12,40]. More generally, the incidence of myopia, particularly progressive high myopia, is higher among females [41]. As an example of how a dominant inheritance pattern of myopia-susceptible opsin alleles might manifest in females, the frequency of G at rs145009674 in *OPN1LW* is 2.1% [33]. Hence, males have a 2.1% chance of having one myopia allele, but females have a 4.1% chance of having one copy and a 0.044% chance of having two copies. Thus, the cone mosaics are different for males versus females with one mutant allele. People of European ancestry, on average, have a ratio of 2L:1M cones; therefore, males have two mutant cones for every normal one. Females with one myopiagenic *OPN1LW* haplotype and one non-myopiagenic *OPN1MW* haplotype have, on average, two normal cones for each one expressing the myopia-susceptible allele. However, if the frequency of mutant and normal cones adjacent to one another is important, not the number of cones expressing the myopia-susceptible allele, then males and females who inherit one myopia-susceptible allele can be similarly affected.

As shown in Table 1, some haplotypes with the highest myopia risk do not have high exon-3 skipping values; this is not unexpected. For example, MVVIS exhibits 0% exon-skipping, but 13 of 23 subjects with this haplotype have an *OPN1MW* exon haplotype (MVVVA or MVAVA) that displays ≥16% exon-3 skipping. Thus, the high-myopia risk associated with *OPN1LW*_MVVIS_ may be due to its tendency to occur with exon-skipping *OPN1MW* haplotypes. In addition, the genetic polymorphisms affect both the splicing and amino acid codes. Altering the amino acid sequence affects opsin function, which may produce contrast between adjacent cones expressing different haplotypes [14].

### 4.2. Why Was OPN1LW Not Identified in GWAS Searches for Myopia Genes?

Why was *OPN1LW* not identified in GWAS searches for myopia genes [10,42,43,44,45,46]? The failure is not surprising, given that the *OPN1LW*_LVAVA_ haplotype’s role in myopia was overlooked for over 10 years despite direct mapping of MYP1 to Xq28 [40,47,48,49,50,51,52]. Moreover, few GWAS included the X-chromosome, none included opsin gene SNPs, and GWAS relies upon high linkage disequilibrium between marker SNPs and disease-causing mutations [53], but *OPN1LW* exon 3 exhibits a high recombination rate and lower-than-expected linkage disequilibrium [19]. Finally, the genes vary in copy number, but only two genes per array are expressed, and GWAS could not eliminate the non-expressed genes from the analysis.

### 4.3. An Optimized Version of the Myopia Reducing Spectacles

Based on the encouraging results of the eyeglasses study described here, we subsequently further optimized the lens design for efficacy, tolerability, and long-term wear by children. Optimizations included improving the method for incorporating durable light scattering elements, adding a ~7 mm clear central aperture, and excluding the tint [54]. These lenses are now the subject of a controlled, multi-center clinical trial in which 256 myopic subjects were randomized at 14 clinical sites and dispensed lenses comparing myopia progression in children wearing standard-of-care lenses versus two different contrast-reducing lenses that differ in the density of light scattering elements (ClinicalTrials.gov Identifier: NCT03623074). The 12-month interim analysis results demonstrated superiority for cycloplegic SER change from baseline (*p* < 0.0001) for both test arms. One test arm reduced myopia progression by 74%, and the other reduced it by 59% [29,55]. This validates the effectiveness of contrast reduction in reducing myopia.

### 4.4. An Unorthodox Theory of Myopia

The three main results reported here are surprising. First, haplotypes at the *OPN1LW/OPN1MW* locus are *THE* major heritable risk factors for myopia with both large effect sizes and a high frequency in the population, accounting for at least 4.9% of the variance in refractive error. Second, having a submosaic of mutant cones intermixed with normal ones is associated with myopia. Third, spectacles that reduce contrast are associated with a dramatic reduction in eye growth.

What is the mechanism by which these *OPN1LW/OPN1MW* mutations cause myopia? Studies of primate retina circuitry have revealed horizontal cell feedback associated with L and M cones that makes the cones and, in turn, their midget bipolar cells contrast detectors [14,56,57]. Midget bipolar cells signal contrast if the number of quanta caught by the single-cone receptive field center is greater (ON bipolars) or less (OFF bipolars) than the average for the cones in their surroundings. If the single cone in the center has a reduced amount of photopigment, it catches fewer photons than the average for the surroundings, which contains a mixture of normal and mutant cones. Similarly, if the central cone contains a normal amount of photopigment, it catches more photons than the average of the surrounding cones. This suggested to us that the bipolar cells in a person with a submosaic of mutant cones constitutively signal contrast even if the affected person is looking at a white wall with no contrast. If true, then surprisingly, it suggests that contrast signaling by the bipolar cells is a signal for the eye to grow.

We acknowledge that this “contrast theory” of myopia contradicts the orthodox view that blur stimulates eye growth [58,59] and that spatial contrast slows eye growth and prevents myopia [60,61,62,63]. We propose the simple evidence-based hypothesis that, in human children, contrast signaling in the bipolar cells is a signal for the eye to grow and reduction in contrast signaling slows eye growth.

The striking results presented here stand on their own, and the details of arguments for and against various theoretical explanations are beyond the scope of the work presented here. Nonetheless, we will briefly outline the contrast theory in light of the new genetic data.

First, given the nature of bipolar signaling outlined above, the opsin mutations associated with high myopia and common myopia support the contrast theory of myopia. So does the observation that skewed L:M cone ratios protect against myopia. Moreover, other known optical devices that effectively slow the progression of myopia project multiple optical powers onto the retina, which reduces contrast [64,65].

More broadly, we offer the following argument for why human eyes may have evolved to have contrast-driven eye growth. Young children are far-sighted [66]. Literally, this means that far away images are sharply focused and produce high-contrast images on the retina even when the eye is modestly near-accommodated. Thus, distant scenery filling the peripheral vision is highly focused even under moderate near-accommodation, producing high contrast and stimulating eye growth. The distant scenery goes out of focus with small amounts of near accommodation as the eye elongates, lowering contrast and reducing the stimulus to elongate. The balance between contrast and growth stabilizes at emmetropia, when the eye achieves the optimal length for its focusing power. We argue that this mechanism worked well in outdoor environments where humans evolved, but not in modern environments where near-work, such as reading and the use of screens, fills peripheral vision with high-contrast images, stimulating eye growth inappropriately, particularly in genetically predisposed children.

The blur hypothesis is based partly on form-deprivation experiments [58,67,68], in which strong diffusers on animal eyes in laboratory settings cause myopia. However, Smith et al. [69] recently investigated the effect of brighter outdoor lighting on eye growth. Nonhuman primates wore diffusers under conditions where auxiliary lighting raised the light levels from 15–630 lux to ∼25,000 lux for 6 h during the middle of the daily 12 h light cycle. Most animals tested under these conditions developed form-deprivation hyperopia. Thus, as predicted by the contrast theory, diffusers produce a hyperopic shift in primates under light levels that approach natural outdoor conditions, which is where the mechanism for human refractive development evolved. Our theory is that contrast is the signal for the eye to grow. This predicts that diffusers that drastically reduce contrast should prevent the eye from growing, resulting in hyperopia. Thus, under lighting more closely resembling outdoor conditions, primates develop hyperopia, as predicted by the contrast hypothesis.

Contrast-gain adaptation may explain why strong diffusers cause myopia at low light levels. The visual system increases its contrast gain depending on the amount of contrast on the retina [70]. Under the low light levels of laboratory settings, wearing strong diffusers may cause the retinal circuitry to increase the gain until the noise is transmitted as real contrast. Higher light levels may reduce the noise so that strong diffusers, which dramatically lower contrast, slow eye growth and produce hyperopia, as predicted by the contrast hypothesis.

Even though it is the opposite of the orthodox view, the contrast hypothesis is consistent with the facts of human refractive development. Mild hyperopia, the natural state of refractive development in young children, is outgrown as the eye elongates during emmetropization [66]. Myopia researchers have argued that this fits with the form-deprivation results under the logic that, when looking into the distance, hyperopia blurs images on the retina [71,72]. However, this overlooks three facts. First, local retinal mechanisms mediate ocular growth in primates, and the peripheral retina is the primary mediator of emmetropization [73]. Second, under the natural outdoor conditions in which humans evolved, faraway scenery fills our peripheral vision. Third, distant scenes are focused and produce high contrast in far-sighted children because their tonic accommodation offsets their short axial lengths. As the eye grows, any amount of accommodation blurs the images of distant objects, reducing contrast. Finally, once a child has progressed to a myopic refraction, distant scenes that fill the retina are always out of focus and low contrast, and more so if the eye is accommodated. Thus, for distant objects, there is a clear progression in contrast as the eye goes from hyperopia to myopia. Therefore, consistent with the contrast hypothesis, peripheral contrast is progressively reduced in the tonically accommodated eye as it grows, reducing the signal for further growth.

## 5. Conclusions

To the extent that the contrast hypothesis proves correct, it can be the basis for a new rational, mechanistic approach to myopia management. Identifying myopia-susceptible children through genetic testing and intervening early with spectacles that reduce myopia progression by 74% may allow for the elimination of high myopia, in turn reducing the risk for the vision-threatening eye conditions that are secondary to myopia.

## 6. Patents

Neitz, J.; Kuchenbecker, J.A.; Neitz, M. Ophthalmic Lenses for Treating Myopia. U.S. Patent 10,571,717, Granted 02/25/2020.

Neitz, J.; Neitz, M. Methods and apparatus for limiting eye growth. U.S. Patent 10795181, Granted 05/28/2019.

Neitz, M., Neitz, J., Methods of diagnosing and treating eye-length related disorders. U.S. Patent 10487361, Granted 11/26/2019.

## Figures and Tables

**Table 1:** *OPN1LW* expm 3 haplotypes tested for association with calculated SER.

Haplotype ^1^	Amino Acids ^2^	N ^3^	Median/Mean SER OS ^4^	% Exon-3 Skipping	Split-Halves Rank ^5^
AACGGTGG	MVVVA	12	−3.16/−3.14	16	1
AACGGCAT	MVAIS	30	−1.62/−2.00	3	2.49
AACGGTAT	MVVIS	23	−2.01/−2.57	0	2.51
AACGGCAG	MVAIA	67	−1.05/−1.71	2	4.25
GCGGGCAG	LVAIA	77	−0.97/−1.38	14	4.75
GCGGGCAT	LVAIS	157	−0.99/−1.38	1	6
GCGATCAT	LIAIS	25	−1.10/−1.30	1	7
AAGGGCAT	MVAIS	9	+0.15/+0.51	0	8.99
GCGGGGAT	LVVIS	5	−1.06/−1.04	1	9
GCCGGCAT	LVAIS	4	−0.05/−0.25	8	9.02
GCCGGCAG	LVAIA	4	+0.54/+0.92	2	11

^1^ Haplotypes are nucleotide identities for dbSNP ID numbers: rs94930, rs713, rs731614, rs5986963, rs5986964, rs149897670, rs145009674, and rs155715655, in that order. ^2^ Amino acids indicated at protein positions 153, 171, 174, 178, and 180 using the single letter amino acid code (M = methionine, V = valine, L = leucine, A = alanine, I = isoleucine, S = serine). ^3^ Number of subjects with each haplotype. ^4^ Median and mean spherical equivalent refraction (SER) for subjects with the indicated haplotype are separated by a slash. The SER was calculated for the left eye (OS) for each subject from axial length and corneal curvature measurements made with the Zeiss IOL Master (see Supplementary Data) using the formula given in the Methods section and described in Supplementary Figure S1. ^5^ Ranks are those determined in the split-halves analysis, see Supplementary Figure S2.

## Data Availability

All data generated or analyzed during this study are included in this published article and its supplementary information files.

## Supplementary Materials

The following supporting information can be downloaded at https://www.mdpi.com/article/10.3390/genes13060942/s1: Figure S1, Relationship between spherical equivalent refractive error (SER) with axial length (AL)/corneal curvature radius (CC) radio in adults; Figure S2, Spherical equivalent refraction versus *OPN1LW* gene haplotype ranks; Table S1, Baseline characteristics of eye-glasses study participants; Table S2, Splicing assay results.
