# Supplementary material for: Insight from OPN1LW Gene Haplotypes into the Cause and Prevention of Myopia
Source: Genes (Basel). 2022 May 25;13(6):942. doi: 10.3390/genes13060942 (PMC9222437; doi:10.3390/genes13060942)
Supplement: Supplementary file 1 [file genes-13-00942-s001.zip › genes-1666398-supplementary.pdf]

Supplementary Figures and Tables

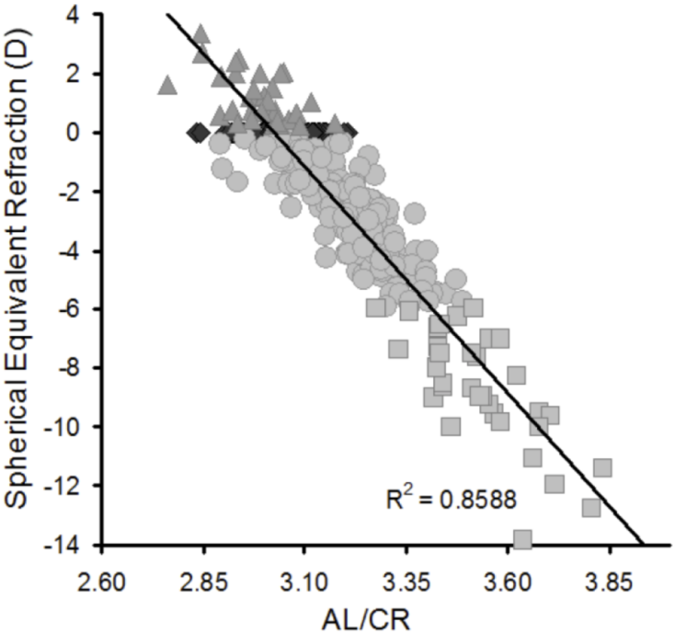

Figure S1. Relationship between spherical equivalent refractive error (SER) with axial length (AL)/corneal curvature radius (CC) radio in adults. Subjects were 17 to 87 years of age (mean age = 36.3 ± 14.9 years, 45% female, 55% male). Refractive error was read from each subject's most recent eye exam (within 6 months) and converted to spherical equivalent refraction (SER = spherical correction + ½ cylindrical correction). Subjects were stratified into four refractive error groups: hyperopes (positive SER, triangles, n = 33), emmetropes (self-reported zero refractive error, black diamonds, n = 152), low to moderate myopes (> -6 D, circles, n = 155), high myopes (≤ -6 D, squares, n = 33). Pearson's correlation (R^2) for the relationship between SER and AL/CC was 0.9. A multiple regression analysis was performed to develop a regression equation for SER. The equation is SER = -(2.03 * AL + 0.94 * CC) + 88.58. The coefficient of determination for comparing SER versus SER predicted by the equation was 0.8644, indicating that the equation accounts for more than 86% of the variation in SER in the adult population.

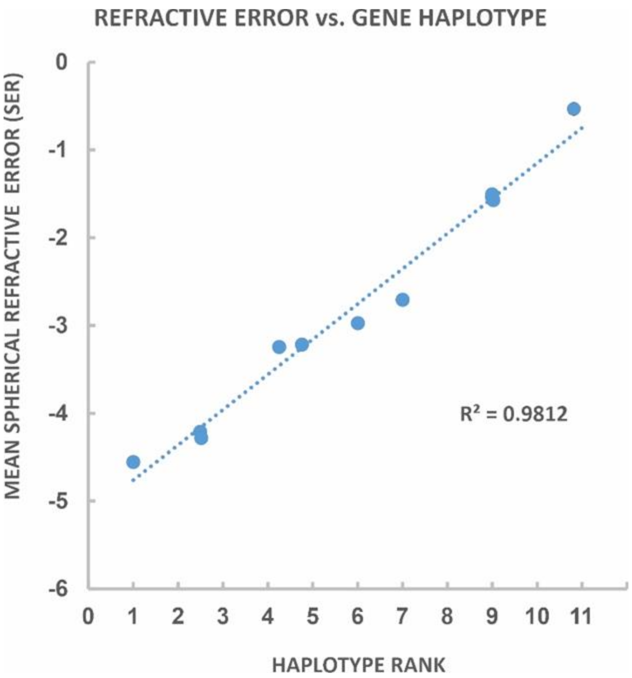

Figure S2. Spherical equivalent refraction versus *OPN1LW* gene haplotype ranks. The X-axis shows the haplotype ranks plotted against each group's mean spherical equivalent refraction from the split halves analysis. The correlation coefficient (R^2) for the association between haplotype rank and refractive error was 0.98, and the probability of this association occurring by chance was $p = 4.51 \times 10^{-9}$. Table 1 lists the haplotypes for each rank plotted.

Table S1: Baseline characteristics of eyeglasses study participants.

Subject ID No.	Gender	Age	SER (OD)	SER (OS)	Axial Length (mm) (OD)	Axial Length (mm) (OS)	Experimental / Control
001	F	12	-1.50	-1.50	25.18	25.27	OD/OS
002	F	13	-3.75	-3.25	24.76	24.95	OS/OD
003	F	14	-7.875	-8.125	27.53	27.84	OD/OS
004	M	11	-3.25	-3.25	23.96	23.93	OS/OD
005	F	9	-2.75	-3.00	24.80	24.86	OS/OD<br>OD/OS
006	F	11	-1.50	-1.375	22.93	23.08	OS/OD<br>OD/OS
007	M	8	-1.50	-1.50	25.25	25.17	OD/OS<br>OS/OD
008	M	13	-1.75	-1.50	24.39	24.54	OD/OS<br>OS/OD
009	F	10	-2.125	-2.125	25.88	25.98	OS/OD<br>OD/OS
010	F	11	-1.375	-1.375	24.15	24.10	OS/OD
011	M	8	-1.50	-1.50	24.08	24.09	OD/OS
012	M	11	-1.125	-1.25	23.21	23.46	OS/OD<br>OD/OS
013	M	12	-4.00	-3.75	25.85	25.77	OS/OD<br>OD/OS

Table S2 Splicing Assay Results

Amino Acid Variant	RS713	RS5986963 RS596964	RS149897670	RS145009674	RS155715655	RS949430(A) RS731614 (C)	RS949430(A) RS731614 (G)	RS949430(G) RS731614 (C)	RS949430(G) RS731614 (G)	SEM, # of replicates[2]
						% exon 3 skipped[1]				
LIAIA	C	AT	C	A	G	8%	7%	7%	11%	
LIAIS	C	AT	C	A	T	1%	1%	3%	1%	
LIVIA	C	AT	T	A	G	10%	26%	18%	37%	
LIVIS	C	AT	T	A	T	1%	3%	1%	5%	
LVAIA	C	GG	C	A	G	12%	9%	13%	14%	1.03%, n = 4
LVAIS	C	GG	C	A	T	2%	0%	2%	1%	
LVVIA	C	GG	T	A	G	1%	3%	3%	8%	
LVVIS	C	GG	T	A	T	0%	0%	1%	1%	0.95%, n =
LIAVA	C	AT	C	G	G	92%	97%	84%	87%	
LIAVS	C	AT	C	G	T	31%	44%	54%	67%	
LIVVA	C	AT	T	G	G	95%	97%	96%	70%	5.07%, n= 4
LIVVS	C	AT	T	G	T	33%	44%	35%	31%	
LVAVA	C	GG	C	G	G	80%	96%	75%	76%	
LVAVS	C	GG	C	G	T	5%	11%	10%	16%	
LVVVA	C	GG	T	G	G	62%	69%	56%	48%	2.16%, n = 4
LVVVS	C	GG	T	G	T	10%	5%	17%	25%	1.25%, n = 4
MIAIA	A	AT	C	A	G	14%	12%	27%	25%	
MIAIS	A	AT	C	A	T	5%	8%	13%	5%	
MIVIA	A	AT	T	A	G	8%	9%	3%	5%	
MIVIS	A	AT	T	A	T	1%	0%	1%	0%	1.0%, n = 3
MVAIA	A	GG	C	A	G	2%	6%	0%	2%	
MVAIS	A	GG	C	A	T	3%	0%	0%	0%	
MVVIA	A	GG	T	A	G	3%	2%	1%	0%	
MVVIS	A	GG	T	A	T	0%	0%	0%	0%	
MIAVA	A	AT	C	G	G	95%	80%	87%	98%	
MIAVS	A	AT	C	G	T	20%	20%	45%	35%	
MIVVA	A	AT	T	G	G	5%	47%	95%	89%	
MIVVS	A	AT	T	G	T	6%	9%	31%	21%	
MVAVA	A	GG	C	G	G	25%	21%	75%	78%	
MVAVS	A	GG	C	G	T	1%	1%	5%	4%	
MVVVA	A	GG	T	G	G	16%	16%	19%	21%	1.08%, n = 4
MVVVS	A	GG	T	G	T	3%	0%	1%	6%	2.02%, n = 4

[1]The percentage of mRNA with exon 3 skipped is given for each haplotype. Haplotypes are given in rows. Nucleotides at SNPs rs713, rs5986963, rs596964, rs149897670, rs15009674, and rs155715655 are given in columns labeled with the SNP IDs, and the corresponding amino acid combination specified is given in column 1. The nucleotides shown for these positions in each row were paired with four different combinations of nucleotides at SNP IDs rs949430 and rs731614 (columns 7-10).

[2]Standard error of the means (SEM) are given. Except where indicated in this column, two biological replicates were performed for each haplotype. The number of biological replicates is indicated by n = x. The percentage of exon 3 skipped in red text indicates the specific haplotype for which more than two biological replicates was performed. For example, for LVAIA haplotype GCGGGCAG, 4 replicates were done, the average was 14% with SEM of 1.03%. See text for single letter amino acid code.
